# Universality of aging: family caregivers for elderly cancer patients

**DOI:** 10.3389/fpsyg.2014.00744

**Published:** 2014-07-15

**Authors:** Lea Baider, Antonella Surbone

**Affiliations:** ^1^Institute Sharett of Oncology, Hadassah University HospitalJerusalem, Israel; ^2^Department of Oncology, Assuta Medical CenterTel-Aviv, Israel; ^3^Department of Medicine, New York University School of MedicineNew York, NY, USA

**Keywords:** cancer, oncology, culture, family caregivers, elderly patients

## Abstract

The world population is aging, with the proportion of older people (65+ years) expected to reach 21% in 2050 and to exceed the number of younger people (aged 15 or less) for the first time in history. Because cancer is particularly a chronic disease of older people, a large increase in the number of elderly patients with cancer is anticipated. The estimated number of new cancer cases worldwide among people over 65 is expected to grow from about 6 million in 2008 to more than 11 million during the coming decade. By 2030, individuals over 65 are expected to account for 70% of all cancer patients in the Western world. Along with the increase in oncology patients, the number of older people caring for their ill spouses or other relatives is also growing, with the ensuing toll on these caregivers causing major concern, especially in western countries. In different societies the characteristics of family caregiver stressors, cultural norms concerning caregiving, and the availability of support have a huge impact on those providing care. Any study of older caregivers of older cancer patients requires an integrative evaluation of aging that takes into account cultural, social, psychological, and behavioral variables. This review proposes a critical discussion of the multidimensionality of the caregiving and of the impact that age, culture, and gender have on it.

## CULTURES OF CARING

Culture may be viewed as a mechanism through which people learn how to “be” in the world – how to behave, how to respect social norms and how to infuse their existence with meaning. Interpretation of culture provides the context for living under free normative conditions in society and across individual lifecycle events (Schim and Doorenbos, 2011).

While Triandis (2007) describes culture as a construct that is still being defined and debated in academic literature and professional disciplines, there is emerging consensus on several variables: (1) culture emerges in interactions between humans and environments; (2) culture enables the sharing of free choices; and (3) culture is transmitted across time periods and generations.

It could be described as “the integrated pattern of human behavior that includes thoughts, communication, actions, beliefs, values, and institutions of an ethnic, religious, or social group” (Cross et al., 1992).

Cultural identification may include, but is not limited to: race, ethnicity, national origin, migration background, gender identity, sexual orientation, marital or partnership status, and spiritual, religious, and political affiliation. Moreover, culture underlies priorities in healthcare services as it is one of the foundations for well-being, survival, human interactions, and quality of life and care (Surbone, 2004).

Dimensions of cultural variables form a background to human interaction across various life stages, and health and illness come to the foreground when people face the realities of an imminent threat to life. The meaning of tradition, rituals, and family values becomes increasingly relevant within the context of illness and health. It is then that patients, families and physicians negotiate the complexity of care within their perception of the reality of each normative culture.

Although much of the extant work on cultural diversity in healthcare has focused on issues of ethnicity, social disparities and religion, the perspective should be broadened to acknowledge differences and similarities between and within groups.

Cross-cultural encounters in healthcare present a challenge with respect to economic, education and health inequalities as well as differences in gender and race (Kagawa-Singer et al., 2010). Acknowledging the profound and pervasive effects of the aforementioned cultural factors, and working toward their mitigation could be essential for solving problems related to equal access to healthcare on the macro and micro level (Schim et al., 2010).

Studies of elderly patients explore the way societal and cultural development affect attitudes toward older people and their family caregivers. In traditional societies old age is often highly valued, with the elderly personifying a source of knowledge and experience. However, in western societies, as the number and percentage of older people increase, they may be seen as a liability and a burden to both their families and society (Surbone et al., 2010).

This is not the case in all cultures, especially non-Western ones, where relatives continue to provide care and total support. At the most fundamental level, perceptions of aging and care are reflections of religious beliefs, traditional family hierarchies, and patriarchal norms of obedience. Attitudes toward caregiving are based in part on cultural and family systems, which shape how caregivers are perceived and whether or not they are treated with respect.

In traditional countries, such as those in the Middle East, the debate concerning knowledge and consent with respect to illness and healthcare revolves around family norms based on the holy books and the family unit is responsible for decisions about treatment and care. These and many other cultures based on community rather than individual rights and responsibilities, value non-maleficence (doing no harm) and strives to protect patients from any emotional and physical harm caused by directly addressing illness and end-of-life care (Searight and Gafford, 2007).

For example, within Islamic culture, the total involvement of caregivers is natural, in the context of the long-held value of respect for unconditional solidarity which is intrinsic to the normative religious-cultural tradition. The integrity and cohesion of relatives as caregivers are understood solely within the dynamic of the family, and not in terms of institutions that provide treatment for illness or terminal care (Baider, 2007).

In the Western world, individual autonomy, truth, and open communication are the core of the dominant bioethical framework. However, the perception of autonomy and openness as empowering and providing a sense of control tends to be blind to the fact that the decision-making process of the individual is involved in a complex relationship with the social surroundings. In Islam, families prefer caregivers to live with uncertainty about the fate of one of its members rather than to confront a poor prognosis or “death threat” (Surbone and Baider, 2013).

## DEMOGRAPHIC OF FAMILY CARE

### FACTS AND FIGURES

The number of older people (65+) across the globe is expected to more than triple by 2100, increasing from 784 million in 2011 to 2 billion in 2050 and 2.8 billion in 2100. The more developed regions of the world have been leading the process of aging and, by 2050, the proportion of older people in these regions is expected to be double that of children (under 15 years): 31.9 vs. 16.3% respectively (World Population Prospects [WPP], 2011).

With a decline in mortality and fertility rates, populations in European countries are aging, leading to the expectation that the demand for healthcare and care for the elderly will rise accordingly. The proportion of individuals aged 65 and over in the 25 member countries of the EU is expected to rise from about today’s 16–30% in the year 2050 (Grammenos, 2005; SHARE, 2012).

Following this trend, populations in developing countries will also be aging rapidly in the coming decades. The number of older people in less-developed countries is expected to increase from 249 million in the year 2000 to 690 million in the year 2030. Since the elderly are at high risk for co-morbidities and disabilities, urgent demands will be placed on developing countries’ healthcare systems – most of which are ill-prepared to handle such pressure.

Inasmuch as cancer is essentially a chronic disease of the elderly, an upward spiral in the number of older individuals with cancer is expected. The incidence of cancer worldwide among people over 65 is expected to grow from about 6 million in 2008 to more than 11 million in the coming decade. By 2030, individuals over 65 will probably account for 70% of all cancer patients in the Western world (SHARE, 2012).

The proportion of malignant tumors, combined with demographic factors, will potentially translate into increasing numbers of diagnoses of cancer, or a high risk for developing cancer (Ferlay et al., 2010).

According to the (Institute of Medicine Report [IOM], 2009), “Family members, friends, and other unpaid caregivers provide the backbone for much of the care that is received by older adults in the United States” – care that is valued, for the year 2007, at ∼$375 billion (National Alliance for Caregiving [NAC] and AARP, 2012). Yet their role is “often underappreciated” and many family caregivers support the elderly patients at significant cost to their own physical, emotional, and financial well-being (National Alliance for Caregiving [NAC] and AARP, 2012).

An estimated 44 million Americans ages 18 and above provide unpaid assistance and support to older people and adults with disabilities who live in the community. Care is provided to someone who is elderly and ill by 65.7 million caregivers who make up 29% of the US population (National Alliance for Caregiving [NAC] and AARP, 2012).

The value of unpaid family caregivers will likely continue to be the largest source of long-term care services in the US, and the aging populations (65+) will more than double from 2000 to 2030, increasing to 71.5 million from 35.1 million in 2000 (Coughlin, 2010).

More women than men are caregivers, with an estimated 66% of caregivers being female. One-third (34%) take care of two or more people, and the average age of a female caregiver is 48.0 (National Alliance for Caregiving [NAC] and AARP, 2012).

Many caregivers of older people are themselves growing older. Of those caring for someone aged 65+ the average age is 63 years with one third of these caregivers in fair to poor health (National Alliance for Caregiving [NAC] and AARP, 2012).

Furthermore, the percentage of caregivers caring for individuals over 85 years of age has increased across all surveys of informal caregivers conducted by National Alliance for Caregiving (1997, 2004, 2009). Parent care continues to be the primary caregiving situation for mid-life caregivers with 70% of the caregivers between the ages of 50 and 64 (Wagner and Takagi, 2010).

A Gallup survey found 72% of caregivers cared for a parent, mother-in-law, or father-in-law, and 67% of caregivers provided care for someone aged 75 or older (Mendes, 2011).

Most care recipients live in their own home (58%) and one in five (20%) live in their caregiver’s home (National Alliance for Caregiving [NAC] and AARP, 2012).

All the preceding facts and figures have social, medical, psychological, economic, and political implications for families and the community as a whole. Society increasingly recognizes that advanced age is a quantitative reality, and that older adults will have to contribute significantly to caregiving for elderly people and aged communities. At the same time, many people face multiple bio-psychosocial challenges as they age. These may be related to physical and cognitive abilities or may be manifest in barriers to accessing mental/behavioral healthcare; decreased economic security; and loss of meaningful social roles and opportunities that allow them to remain engaged in society. These challenges affect entire families, who struggle to provide physical, emotional, financial, and practical support to their aging members.

### CULTURAL COMPLEXITIES: OUTCOMES OF CAREGIVER RESEARCH

Healthcare research clearly indicates that caregiver appraisal should be multidimensional, reflecting culturally competent practice. Studies have shown that cultural beliefs and social norms play a part in influencing family emotions and behavior with respect to cancer perception and in their appraisals of illness and health.

Outcome research shows that access to care may be compromised in some cultures by the family’s reluctance to discuss the disease among its own members, deriving from a belief that “silence should prevail” (Surbone, 2006). Asian Americans believe that talking about death or dying is bad luck, which greatly complicates discussions about prognosis and informed consent. Keeping a cancer diagnosis secret from a patient and avoiding discussions about disease progression can add to a caregiver’s sense of burden and total responsibility. Therefore, early in the initial assessment, cultural beliefs about illness and caregiver roles should be identified and discussed, taking into consideration the family health-culture norms (Ngo-Metzger et al., 2003; Baider and Surbone, 2010).

Multiple social, psychological, and biological factors determine the level of functioning of each caregiver at any point in time. As well as the typical life stressors common to all people, many older adults lose their ability to live independently because of limited mobility, chronic pain, frailty or other mental or physical problems, and require long-term family care. In addition, as previously stated, older caregivers, being themselves elderly, are more likely to experience the drop in socioeconomic status that often accompanies retirement and also chronic illnesses, which may result in isolation, loss of independence, loneliness and psychological distress, all of which add to the caregiver burden.

Studies have been conducted that examine attitudes to older caregivers of elderly patients, and while they tend to be based on surveys in developed countries, there are some that include relevant information from developing countries as well. Studies and anecdotal evidence relating to caregiving in specific national contexts shed some light on the way caregivers are perceived in different countries.

In a meta-analysis of 116 empirical studies, Asian American caregivers were found to provide more caregiving hours than white, African American, and Hispanic caregivers; to use lower levels of formal support services; and to have fewer financial resources, lower levels of education and higher levels of depression than the other subgroups. These findings are relevant for the health-oncology team because caregivers who lack additional medical assistance tend to be more depressed than those who receive support from within the medical team. A study involving unmet needs and service barriers among Asian American caregivers found that caregivers refused outside help because they “felt too proud to accept it” or “didn’t want outsiders coming in.” Other reported barriers included “bureaucracy too complex” or “can’t find qualified providers” (Li et al., 2013).

Hispanic and African American patients and caregivers underutilize community health resources, including counseling and support groups, home care, residential care, and hospice services, one reason being that strong family ties may prevent minority caregivers from seeking help outside the family unit (Guarnaccia and Parra, 1999).

A comparison among African American, white American, and Hispanic caregivers found that 75% of Hispanic patients and 60% of African American patients lived with the family of the primary caregiver. The minority families relied more on informal caregiving from friends and relatives and had larger social support networks than the white families. This increased sense of obligation to provide care for older family members was associated with more caregiving hours, greater resignation about caregiving, higher levels of caregiver strain, and a larger reduction in household income than that reported by white caregivers (Cox and Monk, 1999).

Results showed that African American and Hispanic caregivers were more likely than white caregivers to reduce their work hours to care for sick relatives. In addition, minority caregivers were reluctant to use formal nursing home services for their loved ones or to choose hospice care. The decision to reduce work hours rather than place a relative in a nursing home was associated with increased psychological, social, and financial burden (Covinsky et al., 2001).

Several personal resources that family caregivers draw on – such as their coping strategies, personality factors and social support – are often assumed to be conditioning variables in the caregiving stress process. Rhee et al. (2008) found high depression levels among 66.8% of caregivers for cancer patients, with higher depression levels among women caregivers.

Also among minority groups, caregivers for elderly cancer patients experienced high levels of depression and the presence of depressive symptoms is negatively associated with cancer screening (Park et al., 2013).

### CULTURE, AGE, GENDER, AND CAREGIVING

While traditionally caregiving has been mostly provided by women across cultures, the number of male caregivers is increasing, especially for older males. Most published studies thus far have investigated the effects of caregiving on spouses and women.

Haley et al. (2001) reported that more than 50% of female spousal caregivers showed clinical levels of depression, three times the level found in community samples of people of the same age.

Women caregivers have been found to exhibit moderate to severe sleep problems, fatigue and appetite disturbance, and to report higher levels of cancer-related distress than either cancer patients or survivors. Female caregivers described more unmet needs and a greater sense of burden than male caregivers, leading to the suggestion that male and female caregivers should be regarded as two distinct caregiver groups. This gender difference means women receive less support or acknowledgment for the caring role than do men in the same position (Ussher and Sandoval, 2008).

However, a 13 year follow-up of the Danish population revealed that the same is true for male spouses. Men whose partner was diagnosed with breast cancer were at an increased risk of being hospitalized with an affective disorder compared to men whose partner was not diagnosed with breast cancer (Nakaya et al., 2010).

In a recent study by Goldzweig et al. (2013) psychological distress, social support, and coping strategies reported by partners who were family caregivers to older patients with cancer were compared with those reported by a control group of similarly aged people whose partners were not suffering from life-threatening illness. The results describe relevant implications for caregivers for older cancer patients. Among the research group of caregivers, levels of psychological distress were almost double in comparison to the healthy control group. Caregivers reported lower levels of social support and low levels of coping behaviors which correlated negatively to distress.

Increased ages of patients accentuated caregivers’ distress. Psychological distress of caregivers to cancer patients aged 71+ was four times higher than that of the control group, while they reported half of the coping behaviors reported by the control group. The higher distress levels found in caregivers for older patients could be due to a more realistic sense of inevitable separation and grief. Possibly, caregivers have to deal with feelings of hopelessness and helplessness about their own future as well. Caregivers to older patients (71+) reported lower levels of support from their spouse (the patient) in comparison to caregivers to younger patients (60–70 years). Caregivers for older patients with cancer may experience a continuous process of disengagement resulting in a reduction in social involvement. The emotional support the patient could supply is either diminished or absent, leaving the caregiver with an unfulfilled expectation of support and emotional care.

Why, then, do most family caregivers continue to care for relatives, in spite of all the stress and strain? Research that addresses this question reports that mutual caring can bestow both meaning, through a functional role, and pleasure, on the caregiver and the recipient of care. Kuuppelomaki (2010) found that the most common sources of satisfaction for family caregivers were related to the specific person they were caring for and their ability to provide him or her with an experience of trust and security.

Similar factors emerged as the main satisfaction variables in the study by Nolan and Lundh among family caregivers in the UK and Sweden (Nolan and Lundh, 1999; Sjovall et al., 2009).

Comparable findings came to light in earlier studies concerned with the experiences of stroke, cancer, and dementia patients. Those who care for their relatives at home, often do so of their own volition and a genuine love for the person. In such caring relationships based on love and integrity, the caregiver derives great satisfaction from being able to relieve, bring solace and provide the care recipient with a passionate sense of trust and joy (Pitceathly and Maguire, 2003).

It is crucial to have evidence-based knowledge of the multifaceted aspects of caregiving to suggest possible psychological interventions for caregivers. A recent meta-analysis of different psychological interventions for family caregivers for cancer patients concluded that “clinicians need to deliver evidence-based interventions” to help caregivers and patients to cope effectively, maintain their quality of life, and increase resilience and meaning (Northouse et al., 2010; Stamataki et al., 2014).

The single salient perspective that can be drawn from this overview is that caregiving is not an episodic or closely bound feature of people’s lives. Caregiving involves a long-term commitment over the course of which the conditions that caregivers face, and the transitions through which they must pass are in a state of kaleidoscopic change.

Characteristics of caregiver stressors, cultural norms concerning caregiving, and informal and formal support in different cultures may have a huge impact on caregiver vulnerability and/or resilience. To better understand and ameliorate caregiving stress and focus on evidence-based policies, there is a great need for studies describing appraisals provided by caregivers’ from different cultures about caring for a partner diagnosed with cancer. Specifically, there is a need for studies comparing cultures where most of the care is provided at home by close family members, to cultures where most of the care is provided by professional caregivers outside the patient’s home (Smith et al., 2009).

### PROVISION OF CARE

Caring for an elderly relative who is a cancer patient is extremely complex and demanding. It is described as a blurry mixture of the complex and the simple, of mature and regressive behavior. It is a patchwork of moments of vivid intensity and compassion that at times lapse into blandness and despair.

The caregiver’s life is an oscillation between hope and grief. The close relationship between the family caregiver and the care recipient may require being “on call” 24 h a day, and comprises shared emotions, experiences, and memories that place the caregiver at higher risk for psychological and physical exhaustion (Marks et al., 2004).

Studies consistently report higher levels of depressive symptoms and severe fatigue among caregivers than among their non-caregiving peers (Pinquart and Sorenson, 2003).

Most family caregivers of older people are themselves elderly. The average age of those caring for someone aged 65+ is sixty-three, with one third of these caregivers in fair to poor health. There is cumulative consistent evidence that informal caregivers for elderly cancer patients experience high rates of anxiety and depression, with 20–30% of all caregivers believed to be at high risk for psychiatric morbidity.

Estimates show that between 40 and 70% of caregivers have clinically significant symptoms of depression, with approximately one quarter to one half of these caregivers meeting the diagnostic criteria for major depression (National Alliance for Caregiving [NAC] and AARP, 2008, 2009). In addition, both caregiver depression and perceived burden increase as the care receiver’s functional status declines.

Family caregivers describe feeling frustrated, angry, drained, guilty, or helpless as a result of providing intense care. Some 16% feel emotionally strained and 26% say that caring for the patient is hard on them emotionally. An additional 13% of caregivers feel frustrated when there is little or no improvement in the condition of the care recipient (Center on Aging Society, 2009).

Caregiving may also result in loss of self-identity, lower levels of self-esteem, constant anxiety, or feelings of uncertainty, less self-acceptance, and a sense of being ineffective or lacking control (Buhr et al., 2008).

Furthermore, evidence shows that most caregivers are ill prepared for their role and provide care with little or no support, yet despite this, and the fact that they suffer from poor health themselves, more than one-third continues to provide intense care. It also emerges that an influential factor in a family caregiver’s decision to place a terminally ill relative in a long-term care facility is the state of his or her own physical health (Navaie-Waliser et al., 2004).

Informal family caregivers for cancer patients are required to meet multidimensional needs, including treatment monitoring; treatment-related symptom management; emotional, financial and spiritual support; and the need for assistance with personal and instrumental care. Families are increasingly replacing skilled healthcare workers in the delivery of unfamiliar complex care to their ill relatives, despite the other obligations and responsibilities that characterize their lives (National Alliance for Caregiving [NAC] and AARP, 2008, 2009).

The above data clearly reveals that providing care for a chronically sick person can have harmful physical, mental, and emotional consequences for the caregiver. As family members struggle to care for others, they endanger their own health. Caregiver health is becoming a public health issue that requires more focused attention from health professionals, policy makers and caregivers themselves, to ensure the health and safety of those individuals dedicating their lives to the care of others (Family Caregiver Alliance, 2008).

Increasing appropriate psychological health services and integrative care for family caregivers are appropriate steps toward addressing caregiver health. Although caregiving could have a negative impact on caregivers’ health and well-being, research also demonstrates its effects can be alleviated at least partially by providing adequate skills and professional guidance and organizing community support. Keeping family caregivers healthy and able to provide care is central to maintaining a long-term care system and, with the aging of the population, the issue will only become more significant in the coming decades (Schulz et al., 2004).

Literature on family caregivers of elderly cancer patients highlights: (1) The increasing number of patients with ongoing chronically complex care needs; (2) The increasing number of complex tasks assumed by family caregivers; (3) The high proportion of unmet caregiver needs; (4) The subjective nature of the caregiving experience that encompasses both positive and negative elements, and (5) The conceptualization of caregiver burden as positively linked to negative reactions to caregiving (Stenberg et al., 2011; Li et al., 2013; Milbury et al., 2013).

## CONCLUSION: CREATING A SPACE OF TRUST AND EMPATHY FOR PATIENTS AND CAREGIVERS

Learn from yesterday, live for today, hope for tomorrow. The important thing is to not stop questioning.

Albert Einstein

Any study of caregivers of elderly people requires an integrative evaluation of aging according to cultural, social, and psychological variables. Social norms and traditions regulate the rituals and actions through which people are recognized, and the processes of these dimensions are identifiable within the caregivers’ social and cultural space. Culture also shapes the need for and the behavior of caring, giving it meaning and style in accordance with patients’ values, needs, and priorities.

Age is one of the determinants of the complex notion of culture and, conversely, culture shapes the way we look at the elderly in different societies. The tendency to see older persons through the biased prism of ageism in Western cultures, predominantly based on productivity, is an example of this interrelation (Surbone et al., 2011).

Ageism, by isolating the elderly as no longer productive members of society, determines the individual approach of healthcare providers to older patients and their caregivers, as well as institutional decisions and policymaking in most industrialized countries. By contrast, in other cultures the elderly are considered important members of society, and are respected and valued for their wisdom, so that caregiving is not perceived as only a burden.

Alongside major demographic shifts and globalization there is an increasing multi-ethnicity and multi-culturality in most societies. This calls for the need to learn how to understand, respect and address cultural difference in the practice of medicine and also in sustaining caregivers. Cultural pluralism in individual life, as in medical care, enriches us while also representing a moral and ethical quandary, and misunderstandings, up to real ethical dilemmas, may arise in cross-cultural medical encounters or in communication and interaction with caregivers from different cultures (Surbone et al., 2007).

For this reason, all health workers should acquire “cultural competence” – a set of knowledge, attitudes and skills that facilitates communication and negotiation in cross-cultural clinical encounters with patients and their caregivers. Cultural competence and sensitivity are particularly needed in dealing with elderly patients and their families and caregivers.

Moreover, there is a growing need for more adequately trained healthcare professionals who possess the knowledge and skills to become leaders of the emerging field of geriatrics and the related formal and informal family care (Surbone, 2010).

In the context of this cultural complexity, caring for a family member is not just a private experience, but rather a mutual and interrelated one between caregiver and care recipient within their social and cultural space. When family caregivers hold their ill relatives in their arms they not only fulfill the need for physical contact, but also offer hope and spiritual bonding in a mutual experience of emotional linkage within the boundaries of their traditional behavioral norms.

Surrey (1997) describes the “mutual empathy” of caregiving as an experience of “being with,” and “seeing” the other and sensing his feelings. The outcome of this “seeing” and “being with” is what the caregiver derives from the connection, concern and care. In this mutuality is found a unique experience of communion, meaning, and belonging.

Caregiving is not always supplied by relatives or significant others. We may find caregivers among friends and strangers, in encounters with other families in the hospital or in community settings, as a mutuality of love and concern within a “space” of trust and communion (Baider and Surbone, 2010).

Individuals and systems of care must respond compassionately and effectively to people of all cultures, languages, races, ethnic backgrounds, and religions. Their approach should recognize, affirm, and value individuals, families, and caregivers within their own space, so as to protect and preserve the dignity of each person and family while supporting them through illness and care.

## Conflict of Interest Statement

The authors declare that the research was conducted in the absence of any commercial or financial relationships that could be construed as a potential conflict of interest.
